# Deciphering the molecular regulatory mechanisms of seed oil in soybean for higher-oil breeding

**DOI:** 10.3389/fpls.2026.1964663

**Published:** 2026-10-05

**Authors:** Weitao Xu, Elshan Musazade, Ye Zhang, Moran Bu, Xianzhong Feng, Suxin Yang

**Affiliations:** 1 Key Laboratory of Soybean Molecular Design Breeding, Northeast Institute of Geography and Agroecology, Chinese Academy of Sciences, Changchun, China; 2 College of Advanced Agricultural Sciences, University of Chinese Academy of Sciences, Beijing, China

**Keywords:** higher-oil breeding, oil biosynthesis, regulatory pathway, seed oil content, soybean

## Abstract

Soybean (*Glycine max* [L.] Merr.) is one of the most economically valuable crops globally and a predominant source of vegetable oil. Seed oil content, a core trait that shapes soybean seed quality, is governed by complex polygenic regulatory networks. Identifying key regulators and elucidating the molecular mechanisms of seed oil accumulation thus constitute core research priorities in soybean functional genomics. In recent years, advances in genomics and bioinformatics have substantially accelerated soybean functional genomics research, enabling a more comprehensive understanding of the regulatory networks controlling seed oil content. Here, we summarize recent advances in the molecular regulation of seed oil accumulation, including basal lipid metabolic pathways, central transcription factor regulatory modules, phytohormone signaling, and epigenetic regulation. We also discuss breeding strategies for elevating seed oil content and developing high-oil soybean varieties. Overall, this review integrates the latest findings on soybean seed oil biosynthesis and offers theoretical references for subsequent mechanistic investigations and quality improvement.

## 1 Introduction

Soybean (*Glycine max* (L.) Merr.) is extensively cultivated worldwide and serves as an indispensable source of vegetable oil (Ilangumaran et al., 2022). As a major source of vegetable oil, soybean oil accounts for approximately 27.8% of vegetable oil in the global market (Murphy, 2025). To meet the growing global demand for nutritious food and improved dietary quality, breeding high-quality soybean cultivars has become a major research priority. Cultivated soybeans were domesticated from wild soybeans (*G. soja*) at least 5, 000 years ago through long-term directional selection and genetic improvement (Hyten et al., 2006; Schmutz et al., 2010). They typically contain approximately 20% oil in seeds, which is a core trait determining soybean seed nutritional quality (Wilson, 2004; Wang et al., 2014). Specifically, soybean seed oil consists mainly of five fatty acids (FAs): palmitic acid (16:0), stearic acid (18:0), oleic acid (18:1), linoleic acid (18:2), and linolenic acid (18:3). Accordingly, soybean oil provides essential FAs and features low cholesterol levels. Being readily digested and absorbed, it is effective in combating thrombosis and thus contributes greatly to human health (Ganesan et al., 2018; Petersen et al., 2024). Moreover, soybean oil has broad industrial applications and serves as an important feedstock for the production of nylon, glycerol, biodiesel, and cosmetic products (Dyer et al., 2008; García-González et al., 2008).

The whole-genome sequence of soybean cultivar Williams 82 laid the foundation for soybean functional genomics research (Schmutz et al., 2010). Subsequently, systematic genome sequencing of wild soybean revealed the genetic basis underlying differences between cultivated and wild soybean (Xie et al., 2019). With the rapid development of next-generation sequencing technologies, a wealth of soybean genome resources has been assembled and annotated, covering wild relatives, landraces, and elite cultivated varieties (Shen et al., 2018; Xie et al., 2019; Liu et al., 2020a). Notably, the recent release of telomere-to-telomere (T2T) genome assembly has further filled gaps in centromeric and telomeric regions, greatly improving the integrity of genomic data (Wang et al., 2023). These abundant genomic datasets serve as core foundational resources for comparative genomics research.

Meanwhile, remarkable advances in omics technologies, including transcriptomic, metabolomic, and proteomic analyses, have provided new technical support for functional genomics research on soybean. In recent years, several novel genes responsible for seed oil accumulation have been cloned, and their favorable alleles have been used for quality improvement (Wang et al., 2020; Hu et al., 2023). Different soybean varieties and environmental conditions can lead to significant changes in seed oil content (Wilson, 2004). Therefore, systematic dissection of the hierarchical genetic regulatory network governing seed oil deposition during seed development is urgently required to underpin targeted molecular breeding for soybean seed quality.

In this review, we first summarize the core metabolic processes underlying soybean seed oil biosynthesis and then critically examine the multi-level regulatory mechanisms governing oil accumulation. Unlike previous soybean oil reviews that have largely addressed individual metabolic or regulatory components, this review integrates carbon allocation, FA, and triacylglycerol (TAG) metabolism, transcription factor (TF) networks, phytohormone signaling, epigenetic regulation, and domestication-selected favorable haplotypes within a unified framework. Importantly, we distinguish evidence derived from functional validation, genetic association, and preliminary or heterologous studies to clarify the strength of current mechanistic understanding. Finally, we connect these regulatory insights with breeding-oriented strategies, including precision gene editing and conventional breeding, and highlight key constraints and opportunities for improving soybean oil quantity and quality. Collectively, this integrated perspective links molecular regulation with natural genetic variation and breeding applications, providing a framework for future soybean seed oil improvement.

## 2 Seed oil biosynthetic pathways

Seed oil synthesis is a complex metabolic process involving the coordinated action of multiple organelles and a series of enzymatic reactions, primarily including *de novo* FA biosynthesis, TAG assembly, and oil body (OB) formation (Li et al., 2026a). Thus, the amount of seed oil is regulated at multiple stages during seed development (**Figure 1**).

**Figure 1:**
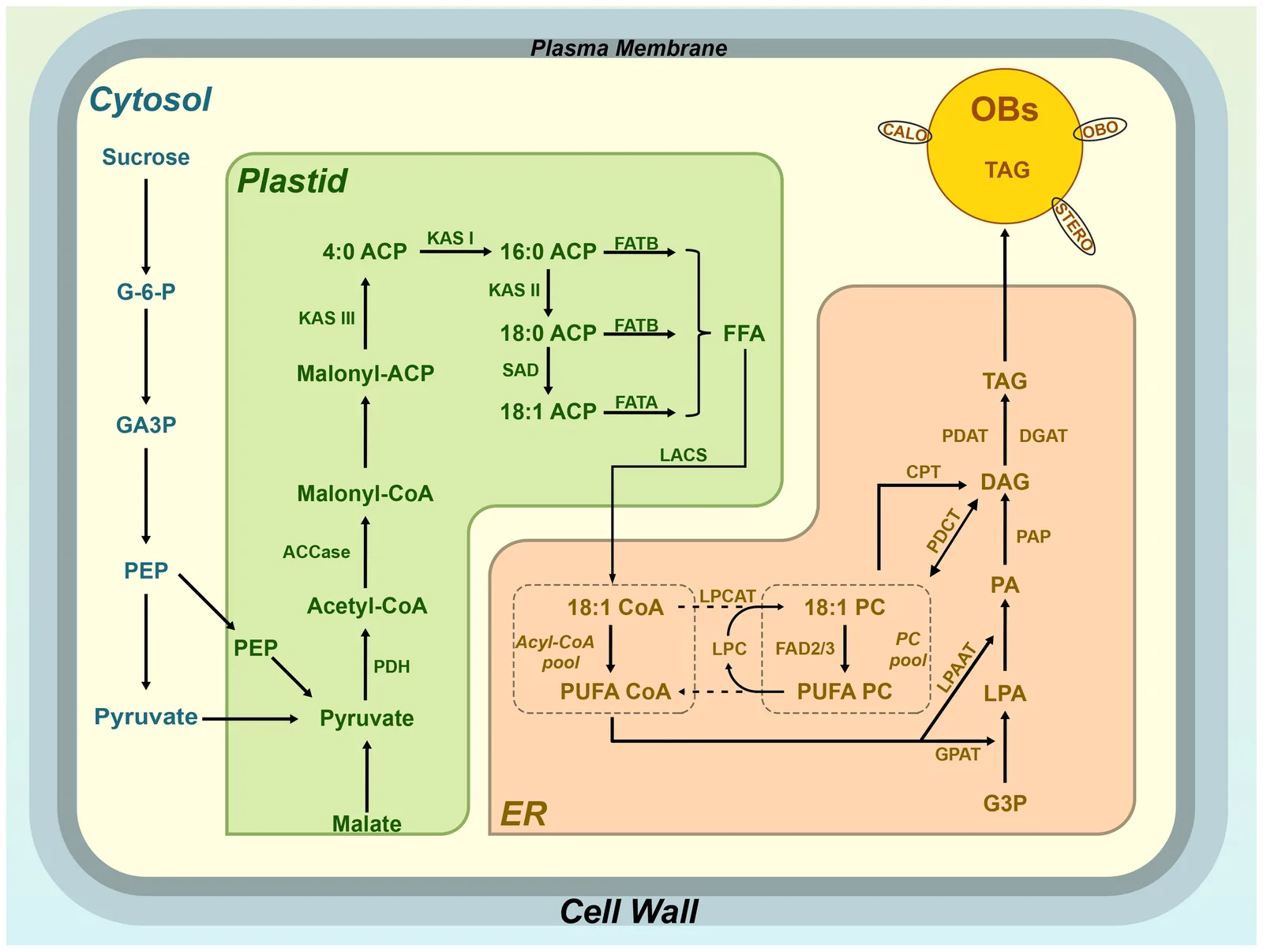
Major metabolic pathways underlying fatty acid (FA) and triacylglycerol (TAG) biosynthesis in soybean seeds. The schematic illustrates carbon flow from sucrose through glycolysis and plastidial FA synthesis, followed by FA modification and TAG assembly in the endoplasmic reticulum (ER) and subsequent storage in oil bodies (OBs). G-6-P, glucose-6-phosphate; GA3P, glyceraldehyde-3-phosphate; PEP, phosphoenolpyruvate; PDH, pyruvate dehydrogenase; ACCase, acetyl-CoA carboxylase; KAS I/II/III, ketoacyl-ACP synthase I/II/III; SAD, stearoyl-ACP desaturase; FATA/B, acyl-ACP thioesterase A/B; FFAs, free fatty acids; LACS, long-chain acyl-CoA synthetase; LPCAT, acyl-CoA:lysophosphatidylcholine acyltransferase; PC, phosphatidylcholine; LPC, lysophosphatidylcholine; FAD2/3, fatty acid desaturase 2/3; G3P, glycerol-3-phosphate; GPAT, glycerol-3-phosphate acyltransferase; LPA, lysophosphatidic acid; LPAAT, lysophosphatidic acid acyltransferase; PA, phosphatidic acid; PAP, phosphatidic acid phosphatase; DAG, diacylglycerol; PDCT, phosphatidylcholine:diacylglycerol cholinephosphotransferase; CPT, CDP-choline:diacylglycerol cholinephosphotransferase; DGAT, diacylglycerol acyltransferase; PDAT, phospholipid:diacylglycerol acyltransferase; OBO, oleosin; STERO, steroleosin; CALO, caleosin; TAG, triacylglycerol.

FAs are synthesized *de novo* in seed plastids, with sucrose produced by photosynthesis serving as the primary carbon source. Pyruvate can be converted into acetyl-CoA, which serves as a fundamental carbon precursor for *de novo* FA synthesis. Plastidic pyruvate is supplied via different metabolic pathways. First, sucrose is converted to glucose and fructose via hydrolysis. Glucose is then broken down into phosphoenolpyruvate (PEP) and pyruvate via glycolysis. The PEP transporter imports PEP into the plastid, where it is converted to pyruvate by pyruvate kinase (Hajduch et al., 2011). Pyruvate can be transported passively or via unidentified transporters directly from the cytosol into the plastids. Malate is a critical supplementary carbon precursor for pyruvate synthesis. It is transported into the plastid through dicarboxylate translocators and decarboxylated to pyruvate by the NADP-malic enzyme (Hajduch et al., 2011; Gerrard Wheeler et al., 2016). Pyruvate dehydrogenase (PDH) then converts pyruvate to acetyl-CoA in the plastid (Chhikara et al., 2018).

Acetyl-CoA undergoes a series of complex enzymatic reactions to produce acyl-ACP. Specifically, acetyl-CoA carboxylase (ACCase) catalyzes the formation of malonyl-CoA from acetyl-CoA (Niu et al., 2015). The resulting malonyl-CoA is subsequently converted to malonyl-ACP, which participates in the initial condensation reaction with acetyl-ACP. This reaction, catalyzed by ketoacyl-ACP synthase III (KAS III), initiates fatty acyl chain synthesis and generates 4-carbon acyl-ACP (C4-ACP) (Li‐Beisson et al., 2013; Lim et al., 2017). Ketoacyl-ACP synthase I (KAS I) then mediates iterative elongation by repeatedly adding two-carbon units, yielding palmitoyl-ACP (16:0-ACP). Within the plastids, 16:0-ACP can be further elongated to stearoyl-ACP (18:0-ACP) by ketoacyl-ACP synthase II (KAS II), and then desaturated to oleoyl-ACP (18:1-ACP) via stearoyl-ACP desaturase (SAD) (Chapman and Ohlrogge, 2012; Li et al., 2026a). Acyl-ACP thioesterase A (FATA) and acyl-ACP thioesterase B (FATB) catalyze the release of free fatty acids (FFAs) from acyl carrier protein (ACP). Long-chain acyl-CoA synthetases (LACS) on the outer envelope membrane of plastids esterify FFAs, generating an acyl-CoA pool (Rawsthorne, 2002; Wang et al., 2012).

The acyl-CoA pool supplies activated acyl substrates for TAG assembly, and TAG biosynthesis takes place on the endoplasmic reticulum (ER) membrane via two independent metabolic pathways. The first is the acyl-CoA-dependent Kennedy pathway. In this pathway, glycerol-3-phosphate acyltransferase (GPAT) catalyzes the acylation of glycerol-3-phosphate (G3P) with long-chain acyl-CoAs as acyl donors, yielding lysophosphatidic acid (LPA) (Frentzen et al., 1983). Subsequently, lysophosphatidic acid acyltransferase (LPAAT) further acylates LPA with acyl-CoAs to form phosphatidic acid (PA). Phosphatidic acid phosphohydrolase (PAP) catalyzes the dephosphorylation of PA to generate diacylglycerol (DAG). Finally, diacylglycerol acyltransferase (DGAT) transfers an acyl group from long-chain acyl-CoA to DAG in the terminal acylation reaction, generating storage TAG (Wang et al., 2012). The second pathway is the acyl-CoA-independent PDAT pathway. Phosphatidylcholine (PC) serves as the core scaffold for the synthesis of polyunsaturated FAs (Chapman and Ohlrogge, 2012). PDAT directly employs unsaturated acyl groups bound to PC to assemble TAG, and this pathway predominantly produces TAG rich in 18:2 and 18:3, which determines the FA composition and quality of seed oil. Acyl-CoA:lysophosphatidylcholine acyltransferase (LPCAT) esterifies oleic acid (18:1) to lysophosphatidylcholine (LPC) to form 18:1-containing PC, which initiates the acyl-editing cycle. FA desaturases FAD2 and FAD3 utilize FAs esterified to the PC backbone as substrates to sequentially generate 18:2 and 18:3. The desaturated polyunsaturated FAs can be released from PC and re-enter the acyl-CoA pool, accompanied by the regeneration of LPC to maintain continuous PC cycling. Meanwhile, PC: DAG cholinephosphotransferase (PDCT) and CDP-choline:DAG cholinephosphotransferase (CPT) catalyze the reversible interconversion between PC and DAG, shuttling polyunsaturated acyl moieties from PC into the DAG pool (Bates et al., 2012). Eventually, phospholipid:DAG acyltransferase (PDAT) directly transfers acyl chains from PC to DAG to drive the formation of TAG (Xu and Shanklin, 2016).

Subsequently, TAGs are packaged into OBs, which are surrounded by a phospholipid monolayer and enriched with amphipathic proteins such as oleosins, caleosins, and steroleosins (Bates et al., 2013). Oleosin is the predominant structural protein of OBs, which governs OB morphogenesis and determines the maximum storage capacity of seed lipids (Tzen and Huang, 1992a; Tzen et al., 1992b). Caleosin stabilizes OBs and balances TAG biosynthesis and degradation (Chen et al., 1999). Steroleosin participates in OB membrane homeostasis and seed lipid signal transduction by regulating sterol metabolism (Tzen, 2012). These three types of amphipathic proteins collectively encapsulate TAG molecules to assemble mature storage OBs.

## 3 Multilayered genetic regulatory networks governing seed oil accumulation in soybean

During seed maturation, the conversion of photosynthetically fixed carbon into storage compounds such as starch, oils, and proteins is tightly regulated by a variety of genes. The final seed oil content in mature seeds is collectively determined by sucrose supply, enzymatic lipid synthesis, transcriptional reprogramming, and multiple upstream signaling inputs, forming an elaborate multilayered genetic regulatory system (Bates et al., 2013).

The fundamental framework of FA and TAG biosynthesis is highly conserved between soybean and the model dicot *Arabidopsis thaliana*, which has long served as a reference for dissecting core lipid metabolic genes. Nevertheless, soybean has evolved lineage-specific regulatory modules and domestication-selected allelic variations that fine-tune seed oil traits (Li et al., 2026a).

In the following sections, we dissect the hierarchical regulatory system for soybean seed oil accumulation. Functional genes identified through forward and reverse genetic screening, together with evidence from multi-omics analyses and functional validation, are synthesized and discussed, encompassing primary carbon and lipid metabolism, central TF modules, phytohormone signaling, epigenetic modifications, and other regulatory factors. The major regulatory relationships are illustrated in **Figures 2**–**4**.

**Figure 2:**
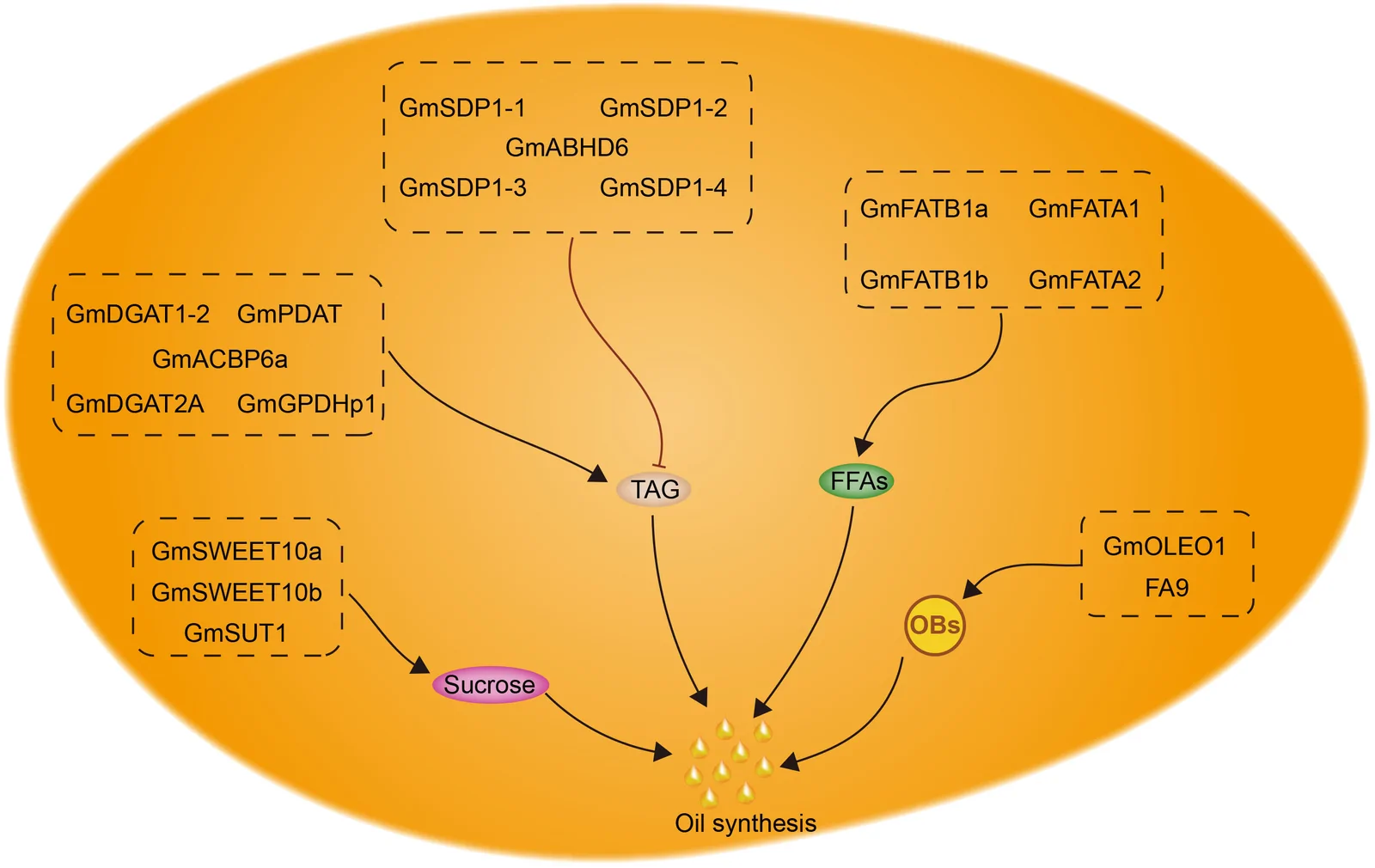
Sucrose supply and core metabolic pathways underlying soybean seed oil accumulation. The schematic summarizes the key functional genes involved in sucrose transport, triacylglycerol (TAG) metabolism, free fatty acid (FFA) production, and oil body (OB) formation, which collectively contribute to seed oil synthesis. Arrows and T-bars indicate positive (activation) and negative (repression) regulatory effects, respectively. The pathway diagrams were synthesized from the published literature and are intended as conceptual regulatory frameworks rather than the results of *de novo* genome-wide orthology analysis or original functional validation.

**Figure 3:**
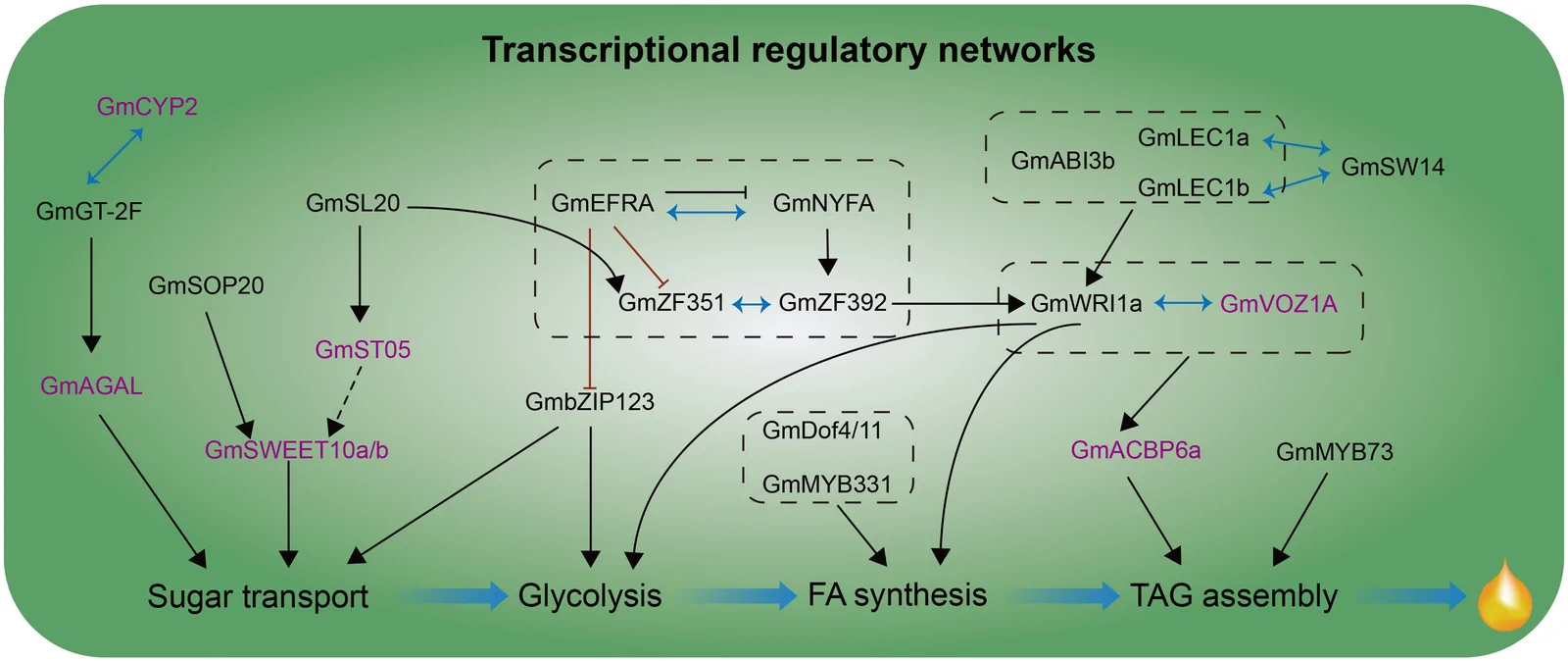
Hierarchical transcriptional regulatory network controlling soybean seed oil accumulation. The schematic summarizes transcriptional regulation across major processes contributing to oil accumulation, including sugar transport, glycolysis, fatty acid (FA) synthesis, and triacylglycerol (TAG) assembly. Arrows and T-bars represent positive (activation) and negative (repression) regulatory effects, respectively, while blue bidirectional arrows indicate protein–protein interactions. Solid lines denote regulatory relationships supported by direct experimental evidence, whereas dotted lines represent putative or unverified relationships. Genes highlighted in purple represent non-transcription factor (non-TF) components. The pathway diagrams were synthesized from the published literature and are intended as conceptual regulatory frameworks rather than the results of *de novo* genome-wide orthology analysis or original functional validation.

**Figure 4:**
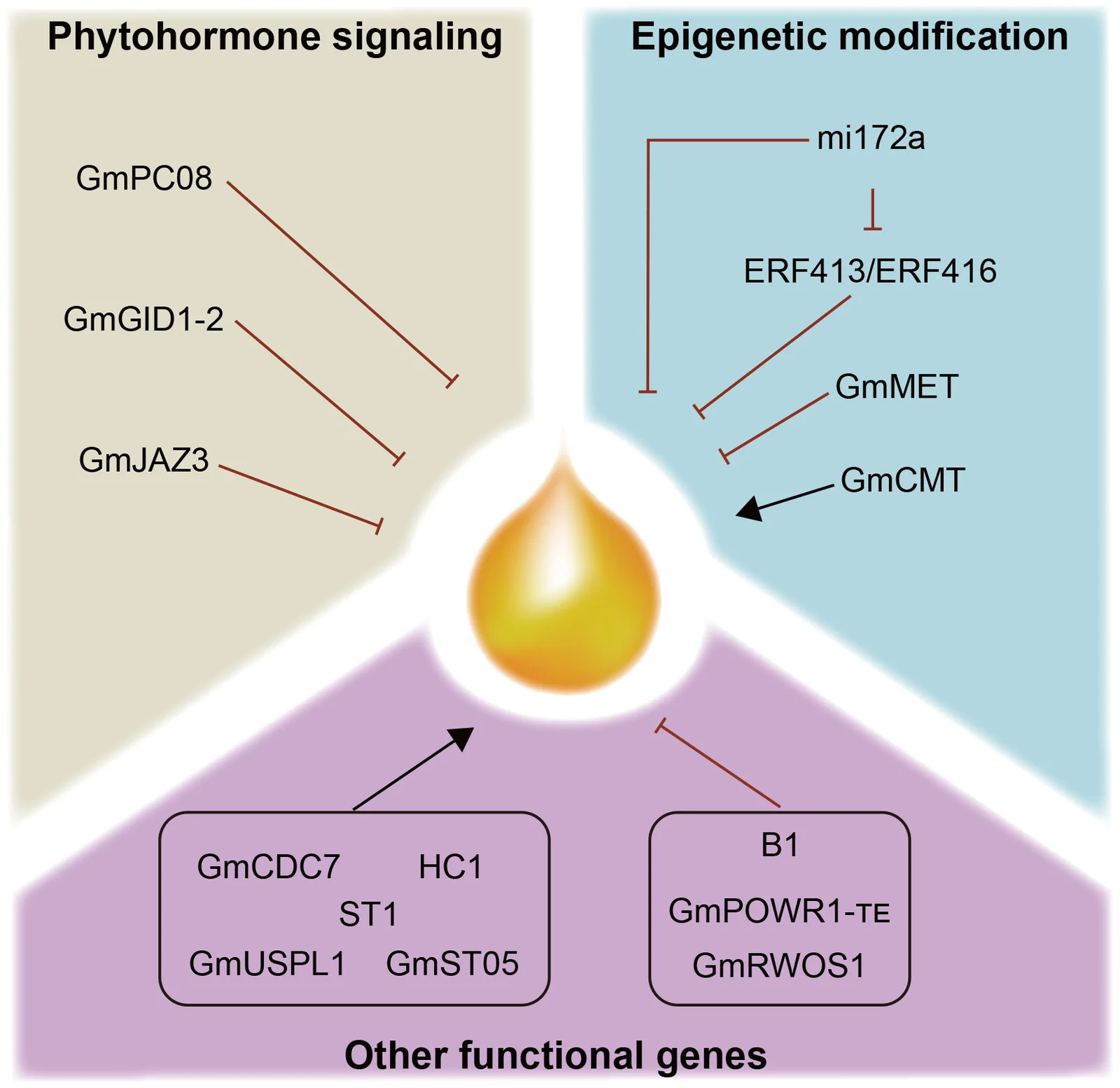
Regulatory pathways involved in soybean seed oil accumulation beyond core lipid metabolism. The schematic summarizes the regulatory components associated with phytohormone signaling, epigenetic modification, and other functional genes that influence seed oil accumulation. Regulatory relationships among the indicated genes and pathways are shown, with arrows representing positive regulatory effects and T-bars representing negative regulatory effects. The pathway diagrams were synthesized from published literature and are intended as conceptual regulatory frameworks rather than the results of *de novo* genome-wide orthology analysis or original functional validation.

### 3.1 Sucrose transport, oil biosynthesis and oil body formation

#### 3.1.1 Sucrose transport

Photosynthate (primarily sucrose) translocation is a key determinant of seed oil accumulation in soybean (**Figure 2**). The sugar transporter genes *GmSWEET10a (Sugars Will Eventually Be Exported Transporter 10a)* and its paralog *GmSWEET10b* modulate seed oil accumulation by mediating intra-seed sugar partitioning (Wang et al., 2020). Knockout of *GmSWEET10a* and *GmSWEET10b* reduces sucrose abundance in mutant embryos, limits FA synthesis, and ultimately decreases seed oil content (Sun et al., 2025). In addition, *GmSUT1 (Sucrose Transporter 1)* exhibits higher expression in the embryos of *GmSWEET10a/10b* double mutants, and this gene exhibits strong and specific expression in the epidermis of the embryos. Knockout of *GmSUT1* via CRISPR/Cas9 led to a pronounced reduction in seed oil content, which recapitulated the phenotypic alterations caused by loss-of-function mutations in *GmSWEET10a/10b*.

These findings collectively indicate that efficient sucrose translocation and partitioning are essential prerequisites for high oil formation in soybean seeds.

#### 3.1.2 FA biosynthesis

Genes encoding core enzymes of oil biosynthesis pathways exert vital regulatory effects on soybean seed oil accumulation (**Figure 2**). As key acyl-ACP thioesterases, FATA and FATB determine the FFAs content in soybean seeds, thereby directly influencing FA levels in mature seeds. The soybean genome harbors two homoeologous FATA genes, *GmFATA1* and *GmFATA2*. Mutations in *GmFATA1* and *GmFATA2* both reduce the total FA content in soybean seeds. In contrast, total seed FA content in the overexpression lines significantly increased, and the enhanced accumulation was mainly attributable to 18:2 (Liao et al., 2024). Similarly, four homologs of *AtFATB*, namely *GmFATB1a, GmFATB1b, GmFATB2a*, and *GmFATB2b*, have been identified in soybean. Knockout of *GmFATB1a* or *GmFATB1b* results in decreased contents of 16:0 and 18:0 without affecting unsaturated FA in the soybean seeds, with more severe phenotypic changes in double mutant lines (Ma et al., 2021). Together, FATA and FATB determine the total pool of free FAs exported from plastids, serving as upstream rate-limiting factors for downstream TAG biosynthesis.

#### 3.1.3 TAG synthesis and degradation

Most FAs synthesized in the plastid are transported to the ER as acyl moieties. Acyl-CoA-binding proteins (ACBPs) facilitate the trafficking of acyl-CoA from the plastid to the ER and participate in TAG biosynthesis (Xiao and Chye, 2011). Overexpression of *GmACBP6a* promotes the accumulation of 18:1 and total oil content in soybean seeds (Yang et al., 2025a).

G3P is an essential substrate for TAG biosynthesis, and its intracellular abundance is a key rate-limiting factor for TAG accumulation (Vigeolas and Geigenberger, 2004). *GmGPDHp1* encodes glycerol-3-phosphate dehydrogenase (GPDH), which catalyzes the biosynthesis of G3P (Zhao et al., 2021). *GmGPDHp1*-overexpressing plants exhibited increased seed oil content and levels of unsaturated FA, particularly 18:1 (Zhao et al., 2021).

DGAT catalyzes the final rate-limiting step of TAG biosynthesis and largely governs TAG accumulation in seeds. Ten *GmDGAT* genes have been isolated in soybean. When all 10 *GmDGAT* genes were transferred into *Arabidopsis*, only *GmDGAT1–2* transgenic lines exhibited higher seed FA content than the wild type. Further analysis showed that overexpression of *GmDGAT1–2* significantly increased 18:1 and total seed oil content, whereas 18:2 declined relative to non-transformant plants (Xu et al., 2021). In addition, another study reported that *GmDGAT2A* positively modulated 18:2 content and total oil content in soybean seeds (Jing et al., 2021). Collectively, different *GmDGAT* paralogs exhibit divergent substrate preferences, jointly shaping total oil content and FA composition in soybean seeds. These results highlight the need to explore the regulatory functions of other *GmDGAT* paralogs in seed oil biosynthesis.

In addition to the DGAT-dependent TAG assembly pathway, the PDAT-mediated pathway serves as an alternative and essential route for plant TAG biosynthesis (Fan et al., 2013). PDAT enzyme activity primarily modulates the FA profile of TAG and participates in seed oil accumulation in coordination with DGAT. *GmPDAT* was identified via a genome-wide association study (GWAS) using 286 soybean accessions (Liu et al., 2020b). Overexpression of *GmPDAT* in *GmPDAT*-OE lines resulted in increased seed oil content, whereas downregulation of this gene in *GmPDAT*-RNAi lines yielded significant decreases in seed oil content (Liu et al., 2020b).

Beyond TAG biosynthesis pathways, TAG degradation and carbon metabolic pathways collectively modulate the final oil concentration of mature soybean seeds by balancing oil synthesis and catabolism. SUGAR DEPENDENT1 (SDP1), a TAG lipase, catalyzes the hydrolysis of TAG. The soybean genome contains four *SDP1* homologous genes (*GmSDP1-1*, *GmSDP1-2*, *GmSDP1-3*, and *GmSDP1-4*), and RNAi-mediated knockdown of these genes effectively suppresses TAG degradation and thereby enhances seed oil content relative to wild-type (WT) plants (Kanai et al., 2019). Similar to SDP1, GmABHD6 (*α/β-Hydrolase Domain-Containing 6*) encodes a monoacylglycerol lipase that negatively regulates seed oil content. Knockout of *GmABHD6* inhibits the hydrolysis of monoacylglycerol (MAG), thereby retaining DAG and TAG and enhancing oil accumulation in soybean seeds. Natural variation in *GmABHD6* further supports its relevance to FA accumulation, as high-FA favorable haplotypes (Hap1 and Hap1-like) were reported to have undergone strong selection during soybean domestication (Yu et al., 2026). As these findings were reported recently, their broader functional significance requires independent validation across additional genetic backgrounds and studies.

It is worth emphasizing that final seed oil content is not solely determined by the capacity of TAG biosynthesis but also by TAG degradation. Specifically, continuous TAG synthesis and catabolic degradation occur simultaneously throughout soybean seed maturation, forming a delicate metabolic balance that ultimately defines the final oil accumulation level. A series of key enzymes and metabolic pathways coordinately regulate this dynamic process. Multiple enzymatic cascades drive TAG synthesis and deposition in lipid droplets (Liu et al., 2020b; Xu et al., 2021; Zhao et al., 2021), whereas lipase-mediated TAG hydrolysis and subsequent fatty acid catabolism promote lipid turnover and can result in substantial oil loss during seed development (Kanai et al., 2019; Yu et al., 2026). This bidirectional metabolic flux means that even enhanced TAG synthesis cannot effectively elevate the final oil content if TAG degradation remains at a high level.

#### 3.1.4 Oil body formation

SEIPIN protein is present in nearly all eukaryotes, where it functions to connect nascent lipid droplets to the ER, stabilizing ER–lipid droplet connections to promote lipid droplet growth (Salo et al., 2016; Wang et al., 2016; Rao and Goodman, 2021). In soybean, a SEIPIN homolog gene was identified as the causal gene within the fatty acid-associated QTL *FA9* (*Fatty acid 9*) via GWAS across 547 diverse soybean germplasm accessions (Qi et al., 2024). Loss of function of *FA9* reduces lipid droplet quantity in soybean seeds and simultaneously decreases seed oil content and unsaturated FA content. The elite haplotype *FA9^H2^* is associated with elevated total FA content in soybean seeds. *GmOLEO1*, an oleosin-encoding gene identified by GWAS for soybean seed oil content, is predominantly expressed at seed maturation stages and specifically localizes to OBs. Overexpression of *GmOLEO1* increases seed oil content by generating enriched smaller OBs (Zhang et al., 2019).

In summary, seed oil accumulation is comprehensively modulated by a series of functional factors at the basal metabolic level. Sucrose transporters regulate carbon source supply and intra-seed carbon partitioning, whereas diverse metabolic enzymes dominate FA biosynthesis and TAG assembly. Structural proteins, including SEIPIN and OLEO1, facilitate lipid droplet biogenesis and stable oil storage. Collectively, these factors constitute the core machinery supporting soybean seed oil biosynthesis, accumulation, and storage.

### 3.2 Transcription factors regulatory networks

TFs play a crucial role in regulating oil biosynthesis and accumulation in seeds (Gazzarrini and Song, 2024). A series of well-characterized TF families governing seed lipid metabolism include APETALA2 (AP2), B3-domain, Nuclear Factor-Y (NF-Y), basic leucine zipper (bZIP), v-myb avian myeloblastosis viral oncogene homolog (MYB), DNA binding with one finger (Dof), Trihelix, and C2H2 zinc-finger proteins. These TFs exert either activating or repressive effects on key regulatory enzymes involved in *de novo* FA synthesis and TAG production, and jointly shape the final oil yield of mature soybean seeds. Below, we systematically summarize the lipid regulatory functions of each core TF family (**Figure 3**).

#### 3.2.1 AP2 TFs

As a core TF modulating seed oil biosynthesis and accumulation, WRINKLED1 (WRI1) is a canonical member of the AP2 family and was first characterized in *A. thaliana* (Maeo et al., 2009). *WRI1* specifically recognizes and binds the conserved AW-box cis-elements in the promoters of its downstream targets to activate gene transcription. These target genes encode essential enzymes involved in glycolysis and FA biosynthesis, including a subunit of pyruvate kinase (*Pl-PKβ1*), acetyl-CoA carboxylase (*BCCP2*), and ketoacyl-acyl carrier protein synthase (*KAS1*). By upregulating the expression of such genes, WRI1 acts as a master positive regulator of seed oil biosynthesis (Maeo et al., 2009). This regulatory function has been validated in multiple plant species, including maize, soybean, and oil palm (Maeo et al., 2009; Shen et al., 2010; Yeap et al., 2017; Zhang et al., 2017; Guo et al., 2020). The soybean genome contains two paralogous *GmWRI1* copies, *GmWRI1a* and *GmWRI1b*. Overexpression of either paralog elevates seed oil content (Chen et al., 2018; Wang et al., 2022; Guo et al., 2020) (**Figure 2**). Nevertheless, these two paralogs exhibit distinct expression patterns during seed development in Wm82 (Zhang et al., 2017). At the R5 stage, the peak period of *GmWRI1* expression, the transcript abundance of *GmWRI1a* is approximately 17.5-fold higher than that of *GmWRI1b*. This expression divergence is attributed to the absence of the RY (CATGCA) cis-regulatory element in the promoter of *GmWRI1b*. The RY motif serves as a binding site for the B3-domain TF GmABI3b, which predominantly enhances *GmWRI1a* transcription and thereby leads to the functional divergence between the two soybean WRI1 paralogs (Zhang et al., 2017).

A recent study has shown that yeast two-hybrid screening in soybean identified vascular plant one-zinc-finger 1A (GmVOZ1A) as a physical interacting protein of GmWRI1a (Yang et al., 2025a). Overexpression of *GmVOZ1A* increases seed oil content in soybean. Comparative transcriptomic analysis further reveals that GmVOZ1A positively boosts seed oil accumulation by modulating the expression of key genes involved in glycolysis, FA synthesis, and TAG metabolism, including acyl-CoA carboxylase (*GmACC*), β-ketoacyl-ACP I synthase (*GmKAS1*), and acyl-CoA-binding protein 6a (*GmACBP6a*). In particular, protein–DNA interaction and dual-luciferase reporter assay revealed that GmVOZ1A and GmWRI1a cooperate to upregulate the expression level of *GmACBP6a* and promote TAG accumulation. In addition, overexpression of *GmACBP6a* promotes the accumulation of seed FA and total oil contents. The identification of this WRI1-interacting cofactor greatly expands the landscape of post-translational regulatory modules that fine-tune WRI1-governed seed oil biosynthesis.

In addition to the positive regulatory branch dominated by WRI1 homologs, the AP2 family contains negative regulators of lipid accumulation. GmERFA acts as a key repressor that reduces seed oil accumulation via dual regulatory mechanisms (Liu et al., 2025a). GmERFA physically interacts with GmNFYA to repress the transcriptional activation of *GmbZIP123* and *GmZF392* mediated by GmNFYA, thereby reducing total oil accumulation in soybean seeds (Liu et al., 2025a). GmERFA also directly suppresses the expression of *GmbZIP123* and *GmZF392*, which are positive regulators of oil accumulation (Song et al., 2013; Lu et al., 2021). The *GmERFA* elite allele carrying the Hap3 promoter haplotype is associated with reduced promoter activity, diminished gene transcription, and increased seed oil accumulation.

#### 3.2.2 B3 TFs and LAFL regulatory module

Plant-specific B3-domain TFs form the core upstream LAFL regulatory cascade, which hierarchically controls *WRI1* transcription and cooperatively promotes seed oil biosynthesis (Wang and Perry, 2013; Elahi et al., 2015; Pelletier et al., 2017). The LAFL module consists of four components: LEAFY COTYLEDON1 (LEC1) (NF-YB family), ABI3, FUSCA3 (FUS3), and LEAFY COTYLEDON2 (LEC2) (the latter three belong to the B3 family). The functional characteristics of *GmLEC1* are elaborated in Section 3.2.3. In contrast to the functionally conserved *GmLEC1* subfamily, soybean lacks functional transcripts of *GmLEC2*, suggesting that *GmLEC2* has likely undergone pseudogenization and lost its canonical role in seed FA biosynthesis. The soybean LAFL members exhibit substantial functional divergence. Only *GmABI3b*, rather than *GmABI3a* and *GmFUS3a/b*, can restore the impaired FA phenotype of the *Arabidopsis lec2* mutant, demonstrating that *GmABI3b* uniquely possesses *LEC2*-like regulatory activity. Collectively, the soybean LAFL network has undergone remarkable functional rewiring. The pseudogenization of *GmLEC2* is accompanied by a compensatory gain of *LEC2*-like function in *GmABI3b*, thereby establishing a distinct regulatory paradigm for seed oil biosynthesis that differs from the canonical *Arabidopsis* network (Zhang et al., 2017). Notably, functional evidence for the LAFL regulatory module remains limited in wild soybean. Whether further evolutionary rewiring of the LAFL module occurred during soybean domestication remains unresolved.

#### 3.2.3 NF-Y TFs

LEC1 encodes an NF-YB family TF. The soybean genome contains four paralogous *GmLEC1* copies. Among them, *GmLEC1a/b* are highly expressed during seed development, whereas *GmLEC1c/d* exhibit extremely low transcript abundance (Zhang et al., 2017). Functional complementation assays in *Arabidopsis* demonstrated that heterologous expression of either *GmLEC1a/b* can effectively restore FA accumulation in the *lec1* mutant, indicating a conserved lipid-regulating function of soybean GmLEC1a/b. Consistently, recent soybean genetic studies further confirmed the essential role of *GmLEC1a* in oil biosynthesis, as its knockout significantly decreases seed oil accumulation (Zhang et al., 2025a). Mechanistically, *GmLEC1a/b* activate the transcription of *GmWRI1a/b* and do not interact with *GmABI3b*.

NF-Y is a protein complex composed of NF-YA, NF-YB, and NF-YC (Myers and Holt, 2018). Given the lipid-regulatory activity of the NF-YB-type LEC1, other NF-Y subunits may also be involved in regulating lipid biosynthesis (Yamamoto et al., 2009). Transcriptomic analyses revealed a correlation between NF-YA expression and oil content during seed development. Overexpression of *GmNFYA* significantly increased FA content in soybean seeds (Lu et al., 2021). The tandem CCCH zinc finger proteins, *GmZF351* and GmZF392, act directly upstream of *GmWRI1* to activate key enzyme genes involved in FA biosynthesis and storage (Li et al., 2017; Lu et al., 2021). *GmNFYA* directly binds to the promoter of *GmZF392*, thereby regulating its expression. GmZF392 interacts with GmZF351 to coordinately regulate downstream genes. Together, they form a novel regulatory module, *GmNFYA*-*GmZF392*-*GmZF351*, and comprehensively coordinate processes from sugar transport and glycolysis to *de novo* FA biosynthesis, TAG assembly, and ultimately OB formation (Lu et al., 2021). Notably, combined GWAS and linkage analyses identified an NF-YA gene designated GmSW14, which directly interacts with the key seed development regulator GmLEC1a/b and suppresses their function to negatively regulate seed oil content (Zhang et al., 2025a).

#### 3.2.4 bZIP TFs

Two bZIP TFs, GmbZIP123 and GmSL20, function as positive regulators of soybean seed oil accumulation. *GmbZIP123* overexpression mutant plants exhibit higher sucrose levels and lipid content in *Arabidopsis* seeds (Song et al., 2013). *GmSL20 (Seed Length 20)* is a TGA-type bZIP TF identified via GWAS (Luo et al., 2026). Knockout of *GmSL20* suppresses seed lipid biosynthesis in soybean, whereas increased expression of *GmSL20* improves seed oil content. Mechanistically, DAP-seq, electrophoretic mobility shift, and transient dual-luciferase assays revealed that GmSL20 directly binds to the promoter and then activates the expression of *GmST05* and *GmZF351*, promoting sufficient carbon substrates for lipid biosynthesis. Haplotype analysis revealed that *GmSL20^H2^* possesses significantly higher transcriptional activation activity and is associated with higher seed oil content compared with *GmSL20^H1^*.

#### 3.2.5 MYB TFs

MYB family TFs modulate seed lipid metabolism through distinct regulatory mechanisms. Heterologous assays in *Arabidopsis* showed that overexpression of *GmMYB73* elevated seed oil content (Liu et al., 2014). GmMYB73 interacts with GL3/EGL3 and represses the expression of *GL2*. Although GL2 is well known for its essential roles in trichome and root hair development, recent evidence has revealed that GL2 negatively regulates seed oil accumulation (Shen et al., 2006). However, these observations provide preliminary heterologous evidence rather than demonstrating conservation of the *Arabidopsis* seed regulatory pathway in soybean seed lipid metabolism, as direct genetic validation of this regulatory module in soybean remains lacking. In contrast, recent direct evidence in soybean showed that *GmMYB331* positively regulates seed oil accumulation (Wang et al., 2026). Mild overexpression of *GmMYB331* increased total seed FA content, whereas CRISPR/Cas9-mediated knockout reduced it. Electrophoretic mobility shift and dual-luciferase reporter assays further showed that GmMYB331 directly regulates *GmAPC4* and several *GmOLEO* family members involved in FA biosynthesis and lipid metabolism. Haplotype analysis additionally identified Hap3 as a favorable promoter haplotype associated with increased seed oil content.

#### 3.2.6 Dof TFs

Dof TFs participate in early FA synthetic pathways. Two characterized members, GmDof4 and GmDof11, promote seed FA accumulation by transcriptionally activating acetyl-CoA carboxylase, the rate-limiting enzyme of *de novo* FA synthesis (Wang et al., 2007). These findings were obtained through heterologous overexpression in *Arabidopsis* and constitute preliminary transgenic data. Functional validation in soybean is still lacking.

#### 3.2.7 Trihelix TFs

A member of the trihelix TFs (GmGT‐2F) regulates source allocation to indirectly control seed lipid traits and positively modulates soybean seed oil content (Li et al., 2026b). *GmGT‐2F* overexpression lines show significantly higher total seed oil, 16:0, 18:1, and 18:2 contents, whereas knockout lines show opposite phenotypes. GmGT‐2F physically binds to the promoter and directly activates the transcription of *GmAGAL*, which encodes α-galactosidase. Single knockout of *GmAGAL* or double mutation of *GmGT‐2F* and *GmAGAL* both markedly reduce α-galactosidase activity and seed oil accumulation, yet no significant phenotypic differences are detected between the two mutant genotypes. Furthermore, the interaction between GmCYP2 and GmGT-2F attenuates the promoter-binding capacity of GmGT-2F, thereby suppressing the expression of *GmAGAL*. Notably, the allelic combination of *GmGT‐2F^Hap2^* with either *GmCYP2^Hap2^* or *GmAGAL^Hap3^* confers superior seed oil accumulation in soybean seeds. This finding awaits independent validation from other research groups.

#### 3.2.8 C2H2 zinc-finger TFs

C2H2 zinc-finger TFs also govern carbon partitioning upstream of lipid biosynthetic pathways. Through GWAS and transcriptome-wide association study (TWAS), GmSop20 (soybean oil and protein QTL on Chr. 20), a key C2H2-type zinc-finger TF regulating the oil content, was identified on soybean chromosome 20 (Zheng et al., 2025). Knockout of *GmSop20* significantly reduces seed oil content, whereas overexpression of *GmSop20* remarkably increases seed oil concentration. The elite haplotype of *GmSop20^C^* upregulates sucrose translocation from the seed coat to the embryo by directly activating the transcription of sucrose transporter genes *GmSWEET10a* and *GmSWEET10b*.

Overall, these diverse TFs form multilayered positive and negative regulatory circuits that coordinate sucrose transport and lipid metabolism at the transcriptional level, acting as the central regulatory hub of seed oil accumulation.

### 3.3 Phytohormones, epigenetic modifications and other functional gene regulatory pathways

In addition to the core metabolic machinery and canonical multilayered transcriptional regulation, seed oil accumulation is further fine-tuned by distal multilayered regulatory pathways that respond to internal developmental cues and external environmental signals. These regulatory modules, including phytohormone signaling, epigenetic modification, and post-translational regulation, indirectly reshape carbon allocation and lipid metabolic flux, thereby optimizing seed oil production at multiple regulatory levels (**Figure 4**).

#### 3.3.1 Phytohormone signaling

Multiple phytohormones form complex signaling cascades that coordinately govern carbon partitioning and seed storage reserve deposition, thereby shaping the final seed oil concentration in soybean. Major phytohormonal regulators, including gibberellin (GA), abscisic acid (ABA), and jasmonic acid (JA), exert effects on lipid biosynthesis (Hu et al., 2023; Liu et al., 2025b; Tang et al., 2026) (**Figure 4**).

The GA signaling pathway plays a core role in plant growth and development (Robil et al., 2025). GIBBERELLIN INSENSITIVE DWARF1 (GID1) functions in GA signaling by interacting with DELLA proteins and GA-dependent repressors and promoting their degradation via the proteasome (Gao and Chu, 2020). GID1 has been shown to regulate pod elongation and seed development in *Arabidopsis* (Gallego-Giraldo et al., 2014). In soybean, recent CRISPR/Cas9-mediated knockout of the soybean GA receptor gene *GmGID1–2* significantly elevated seed oil content, whereas *GmGID1-2*-overexpressing lines exhibited reduced seed oil concentration (Tang et al., 2026). Mechanistically, *GmGID1–2* regulates seed oil accumulation by modulating genes and pathways associated with carbon fixation, alpha linolenic acid metabolism, and linolenic acid metabolism.

ABA signaling is a key regulatory mechanism that determines seed reserve accumulation, and exogenous ABA application can enhance the biosynthesis and accumulation of seed storage compounds (Delisle and Crouch, 1989; Yang et al., 2023). As the rate-limiting enzyme in ABA biosynthesis, 9-cis-epoxycarotenoid dioxygenase (NCED) determines the level of endogenous ABA. In soybean, *GmPC08* (*Protein Contributor 08*) encodes a homolog of *Arabidopsis* NCED5 and functions as a critical negative regulator of seed oil deposition (Liu et al., 2025b). Overexpression of *GmPC08* elevates endogenous ABA levels. Elevated ABA subsequently activates downstream pathways (ribonucleoprotein complex, ribosome, structural constituents of ribosome) that promote storage protein accumulation, which in turn suppresses seed lipid biosynthesis.

In addition to GA and ABA, JA signaling acts as an important regulatory cascade that controls seed lipid biosynthesis, in which the JAZ family proteins function as central transcriptional repressors downstream of JA signal perception. The core gene, *GmJAZ3 (Jasmonate Zim Domain 3)*, was identified through a gene co-expression network of soybean seeds. *GmJAZ3* possesses transcriptional repressor activity, and its overexpression can decrease FA content (Hu et al., 2023). This effect may be mediated by regulating the expression of *GmOLEO1* genes to influence soybean oil content.

GA, ABA, and JA signaling pathways constitute upstream developmental and environmental regulatory layers that influence soybean seed oil accumulation. Despite these phenotypic observations, the regulatory connections between phytohormone signaling cascades, master TFs, and downstream metabolic processes remain poorly characterized in soybean.

#### 3.3.2 Epigenetic modification

Numerous studies have revealed that epigenetic modifications govern diverse seed traits in crops (Xun et al., 2024; Shen et al., 2024; Li et al., 2025). In contrast to stable DNA sequence variations, epigenetic modifications represent sophisticated heritable regulatory mechanisms that modulate gene expression without altering genomic DNA sequences. DNA methylation holds great promise for improving crop productivity and yields, which offers a complementary strategy to conventional genetic approaches (Gallusci et al., 2017). *GmMET* encodes an *S*-adenosyl-L-methionine-dependent methyltransferase that negatively regulates seed oil accumulation (Hu et al., 2026) (**Figure 4**). Seed oil concentration was markedly elevated in *GmMET* knockout lines but substantially decreased in *GmMET* overexpressing lines relative to WT plants. Hypomethylation of *GmSEIPIN1* and *GmNFYA* may be responsible for their elevated transcription and enhanced oil accumulation in mutant seeds. This gene also exerts pronounced effects on unsaturated FA composition. The contents of 18:2 and 18:3 were significantly higher, while 18:1 content was lower in knockout lines compared to WT. By contrast, overexpressing lines exhibited elevated 18:1 and 18:3 contents, yet reduced 18:2 abundance relative to WT. Non-CG DNA methylation, which is primarily regulated by chromomethylases (CMT) and domains rearranged methyltransferase (DRM), is currently known to play important roles in model plants (Hu et al., 2021). In soybean, homozygous lines carrying edits in all four Gm*CMT* paralogs display a significant decline in seed oil concentration (Xun et al., 2024).

Beyond DNA methylation, microRNA (miRNA)-mediated post-transcriptional repression constitutes another independent epigenetic regulatory pathway without altering the underlying genomic DNA sequences. Compared with WT, *MIR172a* overexpression lines showed reduced seed oil content (Jin et al., 2025). 5’ RACE-PCR experiments confirmed that *ERF416* and *ERF413* are target genes of miR172a. Interestingly, *erf416/erf413* mutants exhibited increased FA content. Hereby, miR172a and its downstream ERF416/ERF413 mediate soybean seed oil metabolism through distinct pathways.

Epigenetic modifications, including DNA methylation and miRNA-mediated repression, add a flexible epigenetic dimension to the regulatory network governing soybean seed oil accumulation.

#### 3.3.3 Other functional genes

In addition to phytohormone signaling and epigenetic modulation, a variety of unclassified regulatory proteins further mediate the fine-tuning of soybean seed oil accumulation via distinct signaling and metabolic pathways (**Figure 4**). For instance, *GmST05/MFT (Seed Thickness 05/MOTHER-OF-FT-AND-TFL1)* was identified in soybean via GWAS and belongs to the phosphatidylethanolamine-binding protein (PEBP) family (Duan et al., 2022; Cai et al., 2023). Phenotypic comparison between transgenic lines and WT revealed that seed oil content was significantly elevated in overexpression lines, whereas knockout lines exhibited reduced seed oil levels. The altered seed oil observed in GmST05/MFT-edited plants may be associated with changes in *GmSWEET10a* expression, although the mechanistic relationship between these two genes requires further investigation. Haplotype analysis revealed that high oil soybean germplasms from high-latitude regions predominantly harbor the allelic variants *GmST05^HapI^*/*GmMFT^Hap-H^*.

Transporter-like transmembrane proteins indirectly influence soybean seed oil content by modulating pod seed carbon allocation during domestication and seed development. The *Bloom1* (*B1*) gene controls seed coat bloom formation in wild soybean by functioning in the pod endocarps (Zhang et al., 2018). A natural coding sequence mutation in B1 was artificially selected during domestication, eliminating the powdery bloom on seed coats while simultaneously boosting seed oil accumulation. As a loss-of-function variant, the *b1* allele activates lipid biosynthesis in developing pods and pod endocarps, thereby increasing final seed oil content.

Furthermore, specific carbon metabolism enzymes indirectly regulate seed oil accumulation by modulating glycolytic pathways. *Seed Thickness 1* (*ST1*) was identified via QTL mapping combined with GWAS, and its encoded protein functions as UDP-D-glucuronate 4-epimerase (Li et al., 2022). This enzyme catalyzes the generation of UDP-galacturonic acid, promoting pectin biosynthesis. Further functional evidence indicates that *ST1* positively modulates seed G3P and TAG levels by indirectly affecting glycolysis.

In cereal crops, the CCT (CONSTANS, CO-like, and TOC1)-domain-containing gene is mainly involved in the regulation of photoperiod and grain yield (Liu et al., 2020c). Recently, *POWR1 (Protein, Oil, Weight, Regulator 1)*, a CCT domain-containing gene, was characterized (Goettel et al., 2022). Transposable element (TE) insertion truncates its CCT domain and alters protein nuclear localization, thereby increasing seed oil content. By contrast, transgenic overexpression of *POWR1_-TE_* leads to reduced seed oil content. Mechanistically, *POWR1* modulates seed oil accumulation by orchestrating the expression of key genes associated with nutrient transport and lipid biosynthesis, including *OLEO1*, *DGAT1A*, and *WRI1a*.

*Regulator of weight and oil of seed 1 (GmRWOS1)*, encoding the sodium pump protein, was identified by integrating GWAS and TWAS analyses (Yuan et al., 2024). *GmRWOS1* negatively regulates seed oil content, as demonstrated by gene knockout and overexpression analyses. Meanwhile, natural allelic variants of GmRWOS1 were intensively artificially selected during soybean domestication. *GmRWOS1^Hap1^* exhibits lower expression and higher seed oil accumulation, in contrast to *GmRWOS1^Hap2^*. GmCDC7 (Cell Division Cycle 7) is a typical serine/threonine kinase that positively promotes seed oil levels (Yang et al., 2025b). The *Hilum Colour 1 (HC1)* gene was isolated from soybean through map-based cloning, which positively modulates soybean seed oil levels (Liu et al., 2026). *GmUSPL1 (Universal Stress Protein-Like 1)*, which encodes a BURP domain protein, was identified through the gene co-expression network (Yuan et al., 2024). Knockout of *GmUSPL1* leads to a significant reduction in soybean seed oil content. Although these genes belong to diverse protein families without a unified functional classification, they all participate in the fine-tuning of seed oil levels through independent metabolic or signaling pathways.

Although these genes have been demonstrated to alter seed oil phenotypes, their complete downstream regulatory cascades linking carbon partitioning to FA biosynthesis remain poorly understood, and further mechanistic investigations are required. Collectively, these findings illustrate that soybean seed oil accumulation is controlled by a sophisticated and hierarchical genetic regulatory network. The basal metabolic machinery covering carbon partitioning, FA biosynthesis, and TAG assembly serves as the core executive foundation, whereas diverse TFs act as central switches to modulate lipid gene expression. Furthermore, multiple hierarchical regulatory layers, including phytohormone signaling, epigenetic modification, and various functional regulatory genes, precisely fine-tune metabolic flux at several levels. These layered regulatory mechanisms synergistically coordinate lipid metabolism throughout seed development, jointly determining the final oil yield and FA profile of soybean seeds.

It should be noted that the magnitude of phenotypic effects of the seed oil regulatory genes summarized above is not fixed and can be modulated by soybean genetic backgrounds and growth environment conditions. Nearly all knockout- and overexpression-based functional validations for seed oil related genes have been performed in a limited set of recipient genotypes, predominantly Williams 82, JACK, and DN50 (Zhang et al., 2019; Yang et al., 2025a; Wang et al., 2026). When these genes are introgressed into diverse landraces or elite breeding materials, their impact on seed oil content may be attenuated or enhanced. In addition, environmental factors, including photoperiod, temperature, water, and nutrient supply, can further reshape the final seed oil concentration (Wilson, 2004; Wei et al., 2025). Therefore, even functionally validated favorable alleles require multi-environment field trials across multiple genetic backgrounds prior to large-scale deployment in practical soybean breeding programs.

One of the greatest obstacles to improving soybean seed oil content is the intrinsic trade-off between oil accumulation and seed protein accumulation, as these two traits exhibit a robust inverse correlation. This antagonistic relationship is largely attributed to overlapping biosynthetic pathways and competitive partitioning of carbon and nitrogen resources during seed development (Shi et al., 2025). Traditional soybean breeding frequently encounters a typical dilemma: the genetic enhancement of seed oil content usually leads to a noticeable reduction in protein content, rendering the targeted improvement of oil traits challenging and restrictive (Zhang et al., 2022). Therefore, a primary objective of modern soybean breeding is to precisely elevate seed oil content and optimize fatty acid composition without compromising yield, protein quality, and other vital agronomic traits. Although high-protein soybean varieties require a moderate reduction in oil accumulation, most breeding efforts focus on breaking the oil–protein trade-off to achieve sustainable oil trait improvement (Li et al., 2026a). Future advances in high-oil soybean breeding rely on an in-depth dissection of the genetic, biochemical, and physiological mechanisms underlying oil accumulation, as well as the adoption of precise and innovative breeding strategies to maximize oil yield while maintaining comprehensive field performance.

## 4 Concluding remarks and future perspectives

Seed oil content is a complex quantitative trait controlled by interconnected metabolic, transcriptional, hormonal, and epigenetic networks. Traditional soybean breeding is time-consuming and generally requires multiple generations to establish genetically stable lines, which is labor-intensive. Although hybridization can introduce favorable alleles, linked undesirable alleles may also be co-introduced and require subsequent recombination and selection to eliminate them (Zhang et al., 2025b). Genome-editing technologies, such as CRISPR/Cas9, provide complementary opportunities to accelerate functional validation and precisely manipulate oil-related genes (Zhu et al., 2020). However, their application should be considered in the context of the genetic complexity and extensive gene redundancy of the soybean genome.

Advances in high-throughput sequencing and genetic mapping have substantially expanded the repertoire of genes associated with soybean seed quality traits. Earlier studies frequently focused on soybean homologs of genes characterized in *Arabidopsis* (Liu et al., 2020b), whereas GWAS, QTL mapping, TWAS, weighted gene co-expression network analysis (WGCNA), and multi-omics approaches now enable the more direct identification of soybean regulatory loci and networks (Duan et al., 2022; Zheng et al., 2025; Luo et al., 2026). Nevertheless, association and expression evidence alone cannot establish regulatory causality, emphasizing the need to integrate these approaches with genetic and biochemical validations.

At the metabolic level, genes that control sucrose transport, FA synthesis, TAG assembly, and OB biogenesis collectively determine the carbon flux toward seed oil accumulation (Wang et al., 2020; Liao et al., 2024; Qi et al., 2024; Yu et al., 2026). However, upstream regulators, feedback mechanisms, and functional relationships among multiple isoforms and redundant homologs remain incompletely resolved. Determining whether these homologs function redundantly, synergistically, or in a tissue- and developmental stage-specific manner will be important for identifying effective targets for oil improvement.

A similar knowledge gap exists at the transcriptional level. AP2/EREBP, B3, NF-Y, bZIP, MYB, Dof, Trihelix, and C2H2 zinc-finger TFs participate in the regulation of carbon allocation and lipid metabolism during seed development (Lu et al., 2021; Wang et al., 2022, 2026). However, many reported TF–target relationships remain fragmented across individual studies, and the hierarchical organization of these regulatory networks is not fully established. In addition, high-confidence *in vivo* ChIP-seq evidence remains limited for most soybean seed oil related TFs. Future studies should therefore determine the upstream signals controlling major TF hubs, their direct downstream targets, and the functions of uncharacterized seed-specific paralogs.

Phytohormonal and epigenetic regulation represent another major unresolved layer. GA, ABA, and JA signaling have been implicated in seed lipid metabolism, yet their molecular connections with central transcriptional regulators, such as GmWRI1a, remain insufficiently characterized (Hu et al., 2023; Liu et al., 2025b; Tang et al., 2026). DNA methylation and miRNA-mediated regulation can alter the expression of lipid-related genes (Jin et al., 2025; Hu et al., 2026), but whether many observed epigenetic changes are causal determinants of oil accumulation or downstream consequences of altered seed metabolism remains unresolved. Likewise, several candidates identified through GWAS and population-genetic analyses are associated with seed oil phenotypes; however, their downstream molecular pathways require further functional characterization (Duan et al., 2022; Goettel et al., 2022; Yuan et al., 2024; Yang et al., 2025b).

Total seed oil content and FA composition should be considered as related but partially decoupled breeding targets. Manipulating oil-promoting genes may increase total oil content while producing undesirable changes in FA composition. For example, the overexpression of *GmDGAT1–2* increased the total seed oil and the proportion of 18:1, but decreased 18:2, whereas *GmDGAT2A* positively influenced both the total oil and linoleic acid abundance. Similarly, *GmMET* knockout increased total oil and the proportions of 18:2 and 18:3, while decreasing 18:1. Thus, breeding strategies should simultaneously evaluate oil quantity and FA composition, rather than assuming that increased total oil necessarily improves oil quality.

The second major constraint is the carbon-allocation trade-off among oil, protein, and other seed storage compounds. The carbon imported into developing seeds is finite, and an increasing flux toward TAG synthesis may alter protein or carbohydrate accumulation, depending on the genotype and regulatory context. Genes such as *GmSWEET10a/b* illustrate how regulators of carbon partitioning can simultaneously influence multiple seed components. Consequently, favorable alleles or genome-editing targets should be evaluated not only for oil content but also for their effects on protein concentration, FA composition, seed yield, and other agronomic traits. This is particularly important because pleiotropic effects that improve oil accumulation under controlled conditions may be undesirable at the whole-plant and field levels.

The polygenic nature of seed oil accumulation suggests that manipulating single genes may be insufficient for optimal seed oil enhancement. Rational combinations of favorable alleles could provide a more effective strategy, as demonstrated for multi-allelic breeding approaches in other crops, such as rice (Wu et al., 2018; Ayaad et al., 2020). The favorable haplotypes summarized in **Table 1**, including *FA9^H2^*, *GmSL20^H2^*, and *GmSOP20^C^*, provide potential resources for haplotype-based breeding and allele-pyramiding. However, their effects should be validated across diverse genetic backgrounds and environments before broad breeding programs are implemented.

**Table 1:** Favorable haplotypes associated with soybean seed oil-related traits and their potential applications in breeding programs.

Gene name	Elite haplotype	Source of evidence
*FA9*	*FA9^H2^*	Natural population haplotype analysis + genetic modification verification
*GmSL20*	*GmSL20^H2^*	
*GmABHD6*	*GmABHD6^Hap1_Like^*	
*GmSop20*	*GmSop20^C^*	
*GmST05/GmMFT*	*GmST05^HapI^*/*GmMFT^Hap-H^*	
*GmSWEET10a*	*GmSWEET10a^H_III^*	Natural population haplotype analysis
*GmSWEET10b*	*GmSWEET10b^H_III^*	
*GmMYB331*	*GmMYB331^Hap3^*	
*GmGT‐2F*	*GmGT‐2F^Hap2^*	
*GmAGAL*	*GmAGAL^Hap3^*	
*GmGID1-2*	*GmGID1-2^Pro-C^*	
*ERF416*	*ERF416^Hap3^*	
*GmRWOS1*	*GmRWOS1^Hap1^*	

Haplotypes are classified according to experimental evidence, including those only identified from population-level haplotype association analysis and those further validated by transgenic functional assays.

Several concrete questions emerge for future research. Which combinations of favorable alleles can increase seed oil without reducing protein content or yield? Can elite high-oil haplotypes be pyramided while maintaining desirable FA composition and agronomic performance? Which TFs directly connect GA, ABA, and JA signaling with central lipid regulators such as GmWRI1a? Which paralogous genes have redundant versus specialized functions during seed development? Which GWAS/QTL candidates have causal roles in oil accumulation, and through which downstream pathways do they operate? Finally, how stable are the effects of engineered or naturally favorable alleles across genetic backgrounds, developmental stages, and field environments?

Addressing these questions also requires overcoming the complexity introduced by soybean whole-genome duplication, which has generated extensive paralogous gene redundancy. Such redundancy can mask single-gene knockout phenotypes and complicate CRISPR-based functional analysis, potentially requiring multiplex editing of functionally overlapping paralogs. In addition, genetic modification enables fine-tuning of candidate genes through the use of promoters driving seed-specific spatiotemporal expression, while novel base-editing systems achieve site-specific base substitutions at lipid-relevant genomic sites, collectively expanding the toolkit to enhance soybean seed oil content. Future breeding strategies should therefore integrate multi-omics, causal functional validation, multiplex genome editing, haplotype-based selection, and multi-environment phenotyping rather than relying on individual candidate genes alone. Ultimately, successful improvement of soybean oil traits will require co-optimization of oil quantity, FA composition, protein content, yield, and environmental stability, rather than maximizing seed oil as an isolated trait.
